# UniChem: a unified chemical structure cross-referencing and identifier tracking system

**DOI:** 10.1186/1758-2946-5-3

**Published:** 2013-01-14

**Authors:** Jon Chambers, Mark Davies, Anna Gaulton, Anne Hersey, Sameer Velankar, Robert Petryszak, Janna Hastings, Louisa Bellis, Shaun McGlinchey, John P Overington

**Affiliations:** 1 ChEMBL, Hinxton, CB10 1SD, Cambridge, United Kingdom; 2 Protein Data Bank in Europe, Hinxton, CB10 1SD, Cambridge, United Kingdom; 3 Gene Expression Atlas, Hinxton, CB10 1SD, Cambridge, United Kingdom; 4 ChEBI, EMBL-EBI, Wellcome Trust Genome Campus, Hinxton, CB10 1SD, Cambridge, United Kingdom

**Keywords:** UniChem, InChi, InChiKey, Chemical databases, Data integration

## Abstract

UniChem is a freely available compound identifier mapping service on the internet, designed to optimize the efficiency with which structure-based hyperlinks may be built and maintained between chemistry-based resources. In the past, the creation and maintenance of such links at EMBL-EBI, where several chemistry-based resources exist, has required independent efforts by each of the separate teams. These efforts were complicated by the different data models, release schedules, and differing business rules for compound normalization and identifier nomenclature that exist across the organization. UniChem, a large-scale, non-redundant database of Standard InChIs with pointers between these structures and chemical identifiers from all the separate chemistry resources, was developed as a means of efficiently sharing the maintenance overhead of creating these links. Thus, for each source represented in UniChem, all links to and from all other sources are automatically calculated and immediately available for all to use. Updated mappings are immediately available upon loading of new data releases from the sources. Web services in UniChem provide users with a single simple automatable mechanism for maintaining all links from their resource to all other sources represented in UniChem. In addition, functionality to track changes in identifier usage allows users to monitor which identifiers are current, and which are obsolete. Lastly, UniChem has been deliberately designed to allow additional resources to be included with minimal effort. Indeed, the recent inclusion of data sources external to EMBL-EBI has provided a simple means of providing users with an even wider selection of resources with which to link to, all at no extra cost, while at the same time providing a simple mechanism for external resources to link to all EMBL-EBI chemistry resources.

## Background

There is much data available in the public domain on the structures, effects and interactions of small molecules with biological systems. Many research projects benefit from scientists having easy access to data from these diverse sources. Full data integration (the process of combining data residing within different sources, and presenting the user with a single consistent view) requires that the data models of the different resources be unified in some manner. For resources with very different data models this can be a difficult task, and maintaining the integrated view as data are updated, and underlying data models become modified, can be burdensome.

An alternative to such full-scale integration is to simply provide the user with links or bridges between the separate resources. This alternative suffers from the shortfall of not providing the user with a single point from which all integrated resources can be searched, and requires the user to be knowledgeable of the nature of data likely to be found within these interlinked resources. However, it does nevertheless have significantly lower maintenance costs, and potentially faster performance.

Within EMBL-EBI, there are a number of resources which contain data objects which are small molecules. These include what might be termed primary chemistry-based resources, such as ChEBI [1,2] and ChEMBL [3,4], where small molecules have a central role in their data models, and secondary chemistry-based resources (e.g.: PDBe [5,6], Gene Expression Atlas [7,8]), which have a different main focus (protein structure and gene-expression data, respectively) but which nevertheless are often annotated with small molecule data – for example, the position of a small molecule inhibitor bound to a protein, or the change in gene transcript levels after treatment with a compound. In order to help users make optimum use of all small molecule data at EMBL-EBI, some form of integration solution for all these resources with multifarious data models was considered necessary.

Since these resources are continually developing in response to largely distinct active user communities, a full integration solution, or even the imposition of a requirement to adopt a common unifying chemical identifier, was considered unnecessarily complex, and would inhibit the freedom of each of the resources to successfully evolve in future. In addition, it was recognized that in the future more small molecule-containing databases might reside at EMBL-EBI, either because existing databases may begin to annotate their data with chemical information, or because entirely new resources are developed or adopted. This would make a full integration solution even more difficult to sustain. A need was therefore identified for a flexible integration solution, which would create, maintain and manage links between the resources, with minimal maintenance costs to the participant resources, whilst easily allowing the inclusion of additional sources in the future. Also, since the solution should allow different resources to maintain their own identifier systems, it was recognized as important for the system to have some simple means of tracking identifier usage, at least in the sense of being able to archive obsolete identifiers and assignments, and indicate when obsolete assignments were last in use.

**Figure 1:**
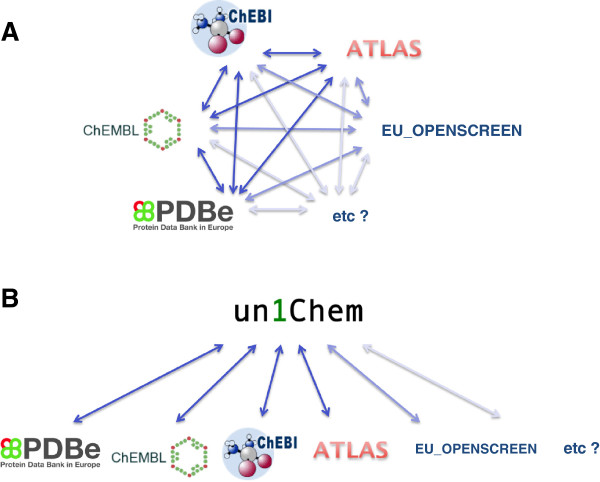
**UniChem efficiently manages the creation and maintenance of structure-based ‘links’ between small molecule containing resources.** Historically, the maintenance of ‘links’ between EMBL-EBI small molecule resources has adopted a model (**A**) where each resource must individually manage its own links to all other resources. The UniChem solution uses a model (**B**) where the mappings are maintained centrally, resulting in significantly lower overall maintenance costs, and allowing for the simple inclusion of additional resources in the future.

Existing resources such as the NIH Chemical Identifier Resolver [9], ChemSpider [10] and the Chemical Translation Service [11,12] all integrate chemical structure related data from a variety of sources, and maintain links between these sources. However, none of these completely fulfilled the current requirements of being able to create complete database to database mappings between EMBL-EBI resources (including the secondary chemistry-based resources referred to above) in a single query, utilizing promptly updated data by an automated ‘pull’ from the source, and track and archive historical identifiers and assignments. Also, some of these are also covered by non-Open licenses, which would preclude independent use. The solution that was developed to address these needs, described here, and made available under a Creative Commons Zero (CC-0) license [13], is termed UniChem. Figure 1 illustrates the process required at EMBL-EBI for linking chemical information across databases before (A) and after (B) the development of UniChem.

The definition of chemical uniqueness in UniChem was an important early stage design decision. The International Union of Pure and Applied Chemistry (IUPAC) International Chemical Identifier (InChI™) is a non-proprietary identifier for chemical substances, specifically designed to be used in printed and electronic data sources in order to facilitate the comparison and linking of diverse chemical data collections. An important feature of the InChI identifier is that it is algorithmically generated by using freely available software maintained by IUPAC and the InChI trust [14]. This enables different groups to independently generate the same InChI for the same structure, and distinguishes the InChI from other chemical identifiers issued by authoritative bodies. In 2009, version 1.02 of the InChI software further improved the utility of the InChI for chemical identifier integration by introducing the ‘Standard InChI’, which does not allow for user selectable options in generating the stereochemical and tautomeric layers of the InChI string. We believe the Standard InChI now largely describes what the community considers to be equivalence between compounds, specifically compounds likely to be of interest in chemical biology and drug discovery. In view of these properties, the standardized form of InChI, and the InChIKey (a 27 character hash code version of the Standard InChI string) were adopted as the chemical structure normalizing key for UniChem.

The overall design of the UniChem database is modeled on UniParc [15,16], which fulfills a similar large-scale, rapid, cross-referencing and archival function for protein sequences. A fundamental feature of UniParc is that the assignment of a sequence to a UniParc identifier is immutable: it is preserved even if the sequence is incorrect, or is deleted from subsequent data releases of the source database. Similarly, for UniChem, each new structure (as defined by the Standard InChI) is assigned a UniChem identifier (UCI) within the system, and this association between the structure and its UCI is never updated or deleted. In an analogous way to UniParc, assignments of source database identifiers to structures are also created, but never deleted, and the only updates permitted are to indicate whether the assignment is ‘current’ or ‘obsolete’. In this way, all structures and assignments of whatever historical status are captured.

## Construction and content

### Database schema

**Figure 2:**
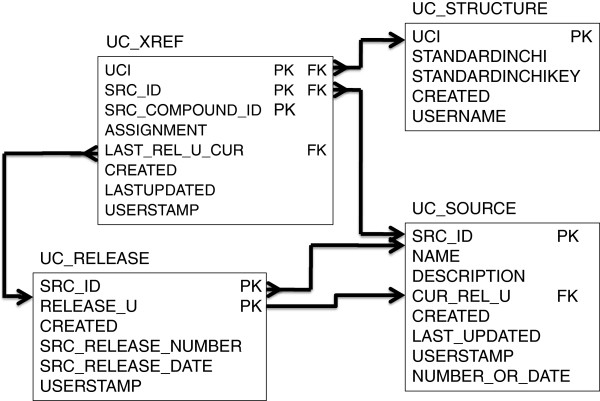
**The UniChem schema.** The UniChem schema consists of four main tables. Structures are stored in the UC_STRUCTURES table, sources in the UC_SOURCES table. The UC_XREF table contains a list of all src_compound_ids to UCI assignments, and fields to indicate whether these assignments are current or obsolete. The UC_RELEASE table tracks information on data releases for all sources. For clarity, not all fields are shown. Primary/foreign key constraints are indicated by solid arrows. PK = Primary Key, FK = Foreign Key.

The schema for UniChem is very simple, consisting of four main tables (shown in Figure 2). In the description of the schema below, schema tables and field names (but not necessarily the variable/identifier names with which they are populated) are shown in upper-case.

Each source database (*e.g*.: ChEMBL, PDBe, etc.) within UniChem is given an identifier (a ‘src_id’), and a single record describing properties of the source is stored in the UC_SOURCES table. The src_id, an integer, is stored in the SRC_ID field, and is the primary key for this table.

All structures in UniChem are held in the UC_STRUCTURES table. Each new structure is assigned a ‘UCI’, which is stored in the UCI field of the UC_STRUCTURES table and acts as the primary key for this table. No records in this table are updated or deleted, only new ones added. Note that the only structural representations in the UC_STRUCTURES table are InChIs and InChIKeys: no Molfiles are stored in UniChem.

The small molecule identifiers, as defined and provided by the individual sources, are termed ‘src_compound_ids’ within UniChem and are stored in the SRC_COMPOUND_ID field of the UC_XREF table. This table contains all current and obsolete assignments of these src_compound_ids to the structures (via the UCI field, which is a foreign key to UC_STRUCTURES.UCI). Note that the UniChem data model allows different sources to use identical src_compound_ids. Therefore to distinguish these identifiers unambiguously the src_id for the identifier must always be specified when referring to a src_compound_id. For this reason, the primary key of the UC_XREF table is a composite of src_compound_id, src_id and UCI. The assignment of a src_compound_id to a UCI in UC_XREF may be flagged as either ‘current’ or ‘obsolete’ in the ASSIGNMENT field. During the loading process, the ASSIGNMENT field may be updated if this assignment has changed from the previous release. Thus if the existing content of this field is ‘current’, but the assignment is no longer present in the newly uploaded data release, then this field will be updated to ‘obsolete’. Correspondingly, if the existing content of this field is ‘obsolete’, but the assignment has re-appeared in the newly uploaded data release (it must have one time been ‘current’ for it to exist at all), then this field will be updated to ‘current’. For assignments that are changed to ‘obsolete’, the LAST_REL_U_CUR field is populated with a ‘release_u’ number, which is an internal release tracking number, and which is a foreign key to the RELEASE_U field of the UC_RELEASE table. The UC_RELEASE table stores information on each data load from each source, and has a compound primary key of UC_RELEASE and SRC_ID fields. Populating the LAST_REL_U_CUR field of the UC_XREF table in this way provides a simple mechanism for tracking the last occasion that an obsolete assignment was current. Clearly, this mechanism cannot fully capture all the possible complexity that may occur over the history of some assignments. Thus, if an assignment were to become obsolete more than once, the complex historical profile of this assignment (e.g.: when it first became obsolete and when it subsequently became current for a second time) would simply not be captured. Such tracking would require a more complex data model than currently exists, and is not considered a sufficiently important requirement based on the lack of use cases for this functionality.

### Sources

Initially, only EBI data sources were used within UniChem, but this has now been expanded to include external sources (e.g.; DrugBank, ZINC). Indeed, any database that contains compounds which have been assigned identifiers and structures, and which makes these data available, can be used as a source within UniChem. The benefits of including additional sources are obvious: all existing and new sources immediately become cross-referenced with each other, all as a result of simply loading the new source data into UniChem.

If Standard InChIs are provided by the source, then these are used directly by UniChem. Unfortunately, not all sources make their structures publically available in the form of Standard InChIs. In these circumstances, UniChem invites such sources to provide, in addition to their preferred representations, Standard InChIs as part of their routine release schedule, so that their source may be simply integrated into UniChem in future. Although the incentive to participate is potentially strong (greater web traffic is likely to be directed towards their resource), we recognize that not all resources may be willing or able to actively participate in this way. Therefore, in a limited number of cases where Standard InChIs cannot be accessed, but where the source is publicly available and deemed to be of sufficient interest, UniChem converts other structural representations (such as Molfiles/sdf, SMILES) into Standard InChIs during the loading process.

### Loading data

Clearly, to populate the UniChem database, data must be downloaded from different sources, then loaded and registered into UniChem. Currently, this is semi-automated but there are plans to have this entire process completely automated. UniChem employs a single uploading and registration process, regardless of the source, in order to maximize the maintainability of the code. However, because the configuration and resources of different source databases vary widely, it is necessary to employ a variety of data downloading procedures (such as ftp, web services, oracle calls, etc.) and source-specific parsers. The downloading procedures adopted for each source are summarized within UniChem, and available for inspection by the user via the ‘sources’ interface page (see below). Currently, all source-specific downloaders and parsers produce a three column data set (src_compound_id, Standard InChI and Standard InChIKey) from each source, which then serves as input for a generic loader.

The UniChem loading process utilizes a number of additional tables (not shown in the schema diagram in Figure 2). These tables serve to optimize the speed of loading, provide some ability to ‘rollback’ loads if necessary, and record various ‘comments’ on loaded data. The loading process is also designed to manage data sets with complex mappings. Thus data sets where multiple src_compound_ids may be assigned to a single Standard InChI, or where single src_compound_ids are assigned to multiple structures, can be managed. In addition, UniChem can record and track changes to these complex mappings from release to release, just as described earlier for simpler mappings.

Since some data are unsuitable for use in UniChem, or might damage the integrity of the database, a series of rules are applied automatically when loading data, and used to filter out unwanted data. A record is not loaded if any of the following 5 rules apply to that record.

1. There is a mis-match between the Standard InChI and the Standard InChIKey.

2. The source providing a list of Standard InChIs does not provide a Standard InChI for a particular record.

3. UniChem cannot generate an InChIKey from the Standard InChI provided by the source.

4. The source does not provide an ID for the structure.

5. The Standard InChI supplied is greater than 2000 characters long.

For most sources, these rules result in the omission of only a very small number of records, but the numbers excluded for the most recent data release from the source are recorded on the individual source pages (see below).

Rules 1 and 3 are important for maintaining data integrity within UniChem. In order to implement these rules, it is necessary for UniChem to generate InChIKeys from InChIs. This is achieved using standard IUPAC libraries for this purpose. In some cases, sources may provide only an InChI but not the corresponding InChIKey. In these circumstances UniChem will calculate the InChIKey and load the record, although noting this absence in a ‘comment’ recorded in the additional loading tables. A small number of the current sources used by UniChem do not provide any Standard InChIs. In these cases, the Molfiles provided by the source have been used and converted to Standard InChIs using the IUPAC InChI generation software. In one case, neither Standard InChIs or Molfiles could be obtained from the source, only SMILES. Since the data was considered to be of particular interest, in this case an additional step was taken to convert SMILES to InChIs using Pipeline Pilot [17]. The length of the cut-off described in Rule 5 was chosen as a suitable length to omit very large molecules from UniChem, and serves to define the meaning of ‘small molecule’ in the context of UniChem.

The frequency with which data is updated in UniChem is optimized for each source as far as is practically possible. Thus for ChEMBL, new data releases occur typically every 3 months, and so ChEMBL data in UniChem is accordingly updated with the same frequency. Tracking of newly available data is easier from sources with defined and versioned release schedules, but other sources can be tracked by other means such as the comparison of download file modification times. However, sources which contain very large amounts of data with very frequent updates (*e.g.*: daily) present a bigger problem, as do sources which do not provide Standard InChIs, since the additional computation required to process and load the data can be considerable. These issues may therefore practically prevent some sources from being updated quite as regularly as may be desired. However, since the dates and versions of the last update are recorded and made visible for each source, the user has the ability to assess whether the update frequencies for their sources of interest are adequate for their purposes.

## Utility and discussion

### Querying options

A user may query UniChem via either a web interface or a RESTful web services API. Both routes provide a range of methods for querying the database in a variety of ways, using four main data types: src_compound_ids, src_ids, Standard InChIs (interface only) and Standard InChIKeys. Querying with a src_compound_id, whether via the interface or the web services, always requires the user to also specify the src_id corresponding to the source from which the src_compound_id originates. This is required because ambiguity of src_compound_ids may exist between different sources. The main features of the interface and web services API are described below. However, many of the specifics of each querying method are not described, as these are fully documented on the UniChem site (https://www.ebi.ac.uk/unichem/), and may be modified in the future in response to user feedback.

Perhaps the simplest question that a user might wish to ask of UniChem is: “Which other databases contain the same structure as src_compound_id ‘x’ from source database ‘y’?”. To answer this question, the user would paste identifier ‘x’ into the main UniChem web interface page, select ‘src_compound_id’ as the data type, select the source database from which this src_compound_id originated (‘y’), and then execute the query. From such a query, the results table will list all the src_compound_ids in all sources that are structurally equivalent to the query src_compound_id (and will include the query term (‘x’) itself). In other fields of the output table, additional information about the structure (the UCI, InChI and InChIKey) and status (“current” or “obsolete”) is given which is frequently useful for a full understanding of the results.

**Figure 3:**
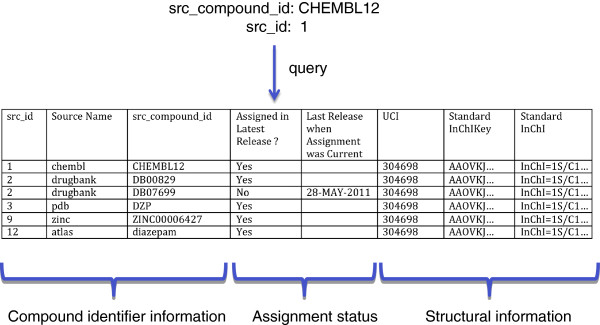
**Example query using the UniChem web interface.** On the UniChem web interface, querying with a single src_compound_id will retrieve a list of all assignments (current and obsolete) which share the same Standard InChI to which the query src_compound_id is currently assigned to. This is illustrated by example in the table below, which shows the data retrieved when querying with the ChEMBL identifier for diazepam: ‘CHEMBL12’. The data columns shown are explained in the text.

An example of this sort of query is shown in Figure 3. Note that in the particular example shown, the structural information is identical for each row. This is because, for simplicity, only a single src_compound_id (‘CHEMBL12’) with a single assignment in UniChem (“assigned only to UCI 304698”) was used to query. Clearly, in this particular example, the structural information is redundant. However, querying with a list of src_compound_ids, or a single src_compound_id with multiple assignments, will produce more complex outputs where structural data will not be identical for each row. In these cases, the structural data will be required for the user to interpret the output correctly: sorting on the structural fields will allow the user to cluster the results according to the individual query src_compound_ids.

Currently, web service API queries using src_compound_ids provide a wider range of search options than the interface method, as described above. One such method is directly equivalent to the above interface query (*i.e.*: all src_compound_ids, whether current or obsolete are retrieved). However, additional API methods may be used to retrieve essentially the same data set as above, but filtered in some way. Thus, one such method allows the user to retrieve only src_compound_ids that are currently assigned to the same structure to which the query src_compound_id is currently assigned to. Using this method, all obsolete assignments are ignored. The result of such a query would therefore retrieve all the records shown in Figure 3, except the obsolete assignment to DB07699. If required, this same API method can be modified to retrieve only src_compound_ids from a defined source. This is achieved by appending an additional parameter to the REST query (*i.e.*: the src_id for the particular sources of interest). Thus, appending ‘2’ to the query for the above example would result in only one record being retrieved (DB00829). Yet another API method can be used to achieve the same result again, but this time returning the full URL for the src_compound_id (if the source supports src_compound_id-specific URLs on their resource). A typical use case for this last query might be where a web manager for a particular resource may wish to construct links from compound pages within their resource to corresponding pages in other resources, by creating on-the-fly web service calls to UniChem each time their compound page is viewed.

Currently used sources in UniChem are listed on a separate page on the web interface, where the src_id corresponding to a particular source may be found. More detailed information on the source, how its data has been processed, and whether it supports src_compound_id-specific URLs (see previous paragraph), may be found by following links on this page. If compound-specific URLs may be created for a source, then the ‘base’ URL (*i.e.*: the URL without the src_compound_id appended) is shown. The same data may be retrieved using the web-services: separate API methods exist to return a list of all valid src_ids, and another, accepting a single src_id as a parameter, will return detailed information on each source.

Searching with structures is also possible. The web interface permits querying with Standard InChIs and Standard InChIKeys, returning the same format of tabulated data as for src_compound_id querying, as shown in Figure 1. Currently, the web-services only support searching with Standard InChIKeys, and not Standard InChIs. Similarity or substructure searches on the structures within UniChem would require very large changes to the UniChem data model, and would be a major departure from the original requirements, and is not planned. However, searching with Standard InChI layers and the Standard InChIKey connectivity layer (*i.e.*: not considering stereochemistry) is feasible without a major change to the original data model, and, although not currently supported, is under consideration for the future.

Lastly, both the interface and web services API support a mechanism to obtain a full mapping of all src_compound_ids between two data sources, by defining a “from” data source and a “to” data source. In this sort of query, termed a ‘whole source mapping’ query, the user selects a ‘from’ src_id, and a ‘to’ src_id. The application then returns a mapping of all src_compound_ids in the ‘from’ src_id to the ‘to’ src_id. In the interface, the user may choose to have the results returned to the browser, or downloaded as a compressed text file. A typical use case for these queries might be data managers who wish to hold all mappings from their resource to all other resources in a local table which might be read when compound pages are constructed. Note that using these mapping methods (or any other API method that retrieves src_compound_ids) in combination with the API methods for retrieving source information (above) allows users to convert retrieved src_compound_ids into URLs programmatically, if required. For example, a ‘whole source mapping’ query between ChEMBL and PDBe would produce a table of mappings between the src_compound_ids for these two sources. Then, by querying for PDBe source information from UniChem, the user may obtain the base-URL for compounds in PDBe (‘http://www.ebi.ac.uk/pdbe-srv/pdbechem/chemicalCompound/show/’). Lastly, by prepending this base-URL to all PDBe src_compound_ids in the mapping table obtained in the first query the user obtains mappings from ChEMBL src_compound_ids directly to the web pages within PDBe which contain the matching structure.

### Mapping precision

The occurrence of multiple src_compound_ids assigned to a single Standard InChI is not uncommon in many sources, especially where the means of compound normalization within the resource itself is not via the Standard InChI. Thus, for example, sources such as ChEBI which utilize a non-Standard InChI as the normalization method (and thereby distinguish tautomers, ionization states, etc. as separate entities), will find, for example, that src_compound_ids corresponding to two tautomeric forms of the same molecule will be represented by a single Standard InChI in UniChem. This therefore results in mappings from both of these src_compound_ids to both tautomers in other sources.

This loss of mapping precision is undoubtedly a drawback of adopting the Standard InChI as the key for defining chemical uniqueness in this situation. However, since we believe that for most purposes this standard largely describes what the chemistry community considers to be equivalence between compounds when considering their biological activity in the context of drug discovery, we consider this to be only a minor shortcoming. Clearly, there are a small number of examples where this is not true. However, we believe most users will understand that: a) this minor loss of precision is an acceptable trade off for up-to-date links provided in an efficient and automatic way, and b) structures where biological activity is so crucially dependent upon tautomeric form are likely to be carefully annotated to alert the user to this subtlety.

### Provenance

Many databases have integrated structures from other sources, quite often adding little or no additional annotation to these structures, and sometimes providing no indication of the origin of these structures. The links created by UniChem between these sources will often therefore, include ‘circular’ links. Circular links take the user from one source to another, only to find that the second source simply contains no additional data, other than perhaps a link back to the original source.

To prevent this, one might suggest that UniChem should therefore only include structures from a source if the source is the primary source for the compound. However, there are a number of reasons why UniChem does not do this.

Firstly, maintaining these ‘rules of provenance’ may appear straightforward in a limited number of cases, but with a large number of sources involved in UniChem, attempting to tease out the primary and non-primary sources for each compound would become very complex, and impose a heavy ongoing curation burden on UniChem. Furthermore, it presupposes that information on the origin of compounds within a source is always available. Also, the policies and decisions required to categorize sub-sets of structures within a source as ‘primary’ or ‘non-primary’ are likely to be complex, and possibly contentious, and would probably result in confused and disillusioned users. One such situation might be when a primary source ceases to exists or is no longer publicly available while secondary sources remain accessible. In such a situation it would be difficult to find community consensus on assigning “primary” status to one of the secondary sources.

Secondly, a plausibly common use case for UniChem might be that of a data manager who wishes to create links between only two sources (i.e.: they wish to ignore all other sources in UniChem). Such users would expect to be able to use UniChem to create all cross-references between compounds in the two sources, irrespective of the origin of the compounds, and would not expect certain compounds from a source to have been ‘removed’ from UniChem because they were not considered to have ‘primary’ status in this particular source.

For these reasons, the problem of preventing ‘circular’ links should therefore be the responsibility of the user of UniChem, and not of UniChem itself. UniChem policy is therefore to load all structures from a source; the bespoke filtering and processing of the feed from UniChem, to prevent circular-links and other anomalies, would then be in the hands of the UniChem user.

Of course, to assist users in these tasks, there is no reason why sub-sets of a source could not be loaded as separate sources in UniChem; the sub-sets being defined on the basis of the original source. Thus ‘PubChem_ChEMBL’ might be a typical subset (i.e.: all PubChem [18,19] structures which originally came from ChEMBL). This would assist users who wish to filter out certain sub-sets from a source. For example, many users might wish to exclude the sub-set of compounds from a source which originate from their own database, in order to prevent ‘circular links’. In the current example therefore, ChEMBL might elect to use UniChem to create links to all sub-sets of PubChem, except for those for those where the primary source of the compound is ChEMBL, and possibly also for other sub-sets commonly held by the two sources.

## Conclusions

UniChem was originally developed as a tool to minimize and share the maintenance costs of creating and maintaining electronic links between resources containing chemical information within EMBL-EBI. The early design decision to adopt the Standard InChI as a normalizing key was based on the belief that this standard has become the preferred structural format for defining chemical uniqueness in the context of biological activity. Although the use of this standard has some drawbacks in the current application (*i.e.*: some loss of mapping precision), we believe this is far outweighed by its very widely accepted nature, and by the convenience and efficiency gains that it permits. Also, because it was believed that cost considerations should be no barrier for sources to participate in the UniChem project, the freely available nature of InChIs as a standard was a highly significant factor in the choice of its use in UniChem.

To the end user, the benefits of UniChem are several-fold. Firstly, compound identifier mappings between sources of biological interest are achieved quickly and easily by either simple web interface queries or web services, as is ‘whole source-to-source mappings’; a service not currently available from similar projects. Also, historical information on the use of compound identifiers is also available: allowing users to assess whether particular identifiers from a source have become obsolete. For data-managers, the creation and maintenance of compound-based links between their resource and all other participating resources is simplified to a process of making available their identifier-to-structure data to UniChem, and then querying UniChem appropriately.

From the perspective of UniChem data managers, the strengths of the UniChem model include the ease with which new sources may be added with minimal effort: A new source-specific downloading mechanism and parser is all that is required to add a new source. Also, since compound cross references between sources are all simply dependent upon matching InChIs in the database, the updating of data from one source in UniChem instantly generates, all within the database, up to date links to and from all other sources.

UniChem is a low-maintenance compound identifier mapping service. The recent exposure of UniChem as a freely available service on the internet will allow a wider community of users to enjoy the benefits of this service. It should be noted that the service is provided via the https protocol, thus ensuring secure querying, a feature that may be of importance for some users. We hope that in the future other chemistry-based sources will actively participate in this project by making available compound identifier and structural assignments in a convenient form for UniChem to use (i.e.: as Standard InChIs and Standard InChIKeys).

## Availability and requirements

UniChem may be accessed at the following URL: https://www.ebi.ac.uk/unichem/ and data is freely available from this site, via the web interface or web services, under a Creative Commons Zero (CC-0) license (http://wiki.creativecommons.org/CC0).

## Abbreviations

IUPAC: International Union of Pure and Applied Chemistry; InChI: IUPAC International Chemical Identifier; UCI: UniChem Identifier; EMBL: European Molecular Biology Laboratory; EBI: European Bioinformatics Institute.

## Competing interests

The authors declare that they have no competing interests. The work was funded by a Strategic Award for Chemogenomics from the Wellcome Trust [086151/Z/08/Z]; and the European Molecular Biology Laboratory; and EU-OPENSCREEN.

## Authors' contributions

JC carried out the design and implementation of the database, web interface, web services, parsers and loaders, and is currently responsible for the maintenance of data sets within UniChem. MD carried out deployment. SM and MD contributed invaluable advice on the design and testing of the web services. JPO, SV, RP and JH participated in early discussions on the conception and design, and the provision of some data sets. LB assisted with the preparation of data sets. All authors provided invaluable feedback and advice on the design and implementation of the entire application, and read and approved the final manuscript.

## References

[B1] ChEBI http://www.ebi.ac.uk/chebi

[B2] de Matos P Alcántara R Dekker A Ennis M Hastings J Haug K Spiteri I Turner S Steinbeck C Chemical Entities of Biological Interest: an update Nucleic Acids Res 2010 38 D249 254 10.1093/nar/gkp886 19854951 PMC2808869

[B3] ChEMBL https://www.ebi.ac.uk/chembl

[B4] Gaulton A Bellis LJ Bento AP Chambers J Davies M Hersey A Light Y McGlinchey S Michalovich D Al-Lazikani B Overington JP ChEMBL: a large-scale bioactivity database for drug discovery Nucleic Acids Res 2012 40 D1100 D1107 10.1093/nar/gkr777 21948594 PMC3245175

[B5] PDBe http://www.ebi.ac.uk/pdbe

[B6] Velankar S Alhroub Y Best C Caboche S Conroy MJ Dana JM Fernandez Montecelo MA van Ginkel G Golovin A Gore SP Gutmanas A Haslam P Hendrickx PMS Heuson E Hirshberg M John M Lagerstedt I Mir S Newman LE Oldfield TJ Patwardhan A Rinaldi L Sahni G Sanz-Garcıa E Sen S Slowley R Suarez-Uruena A Swaminathan GJ Symmons MF Vranken WF Wainwright M Kleywegt GJ PDBe Protein Data Bank in Europe Nucleic Acids Res 2012 40 D445 D452 10.1093/nar/gkr998 22110033 PMC3245096

[B7] Gene Expression Atlashttp://www.ebi.ac.uk/gxa

[B8] Kapushesky M Adamusiak T Burdett T Culhane A Farne A Filippov A Holloway E Klebanov A Kryvych N Kurbatova N Kurnosov P Malone J Melnichuk O Petryszak R Pultsin N Rustici G Tikhonov A Travillian RS Williams E Zorin A Parkinson H Brazma A Gene Expression Atlas update–a value-added database of microarray and sequencing-based functional genomics experiments Nucleic Acids Res 2012 40 D1077 D1081 10.1093/nar/gkr913 22064864 PMC3245177

[B9] NIH Chemical Identifier Resolverhttp://cactus.nci.nih.gov/chemical/structure

[B10] ChemSpiderhttp://www.chemspider.com/

[B11] The Chemical Translation Servicehttp://cts.fiehnlab.ucdavis.edu

[B12] Wohlgemuth G Haldiya PK Willighagen E Kind T Fiehn O The Chemical Translation Service–a web-based tool to improve standardization of metabolomic reports Bioinformatics 2010 26 20 2647 2648 10.1093/bioinformatics/btq476 20829444 PMC2951090

[B13] Creative Commons Zero (CC-0) license http://wiki.creativecommons.org/CC0

[B14] InChI Trusthttp://www.inchi-trust.org/

[B15] UniParc http://www.ebi.ac.uk/uniparc

[B16] Côté RG Jones P Martens L Kerrien S Reisinger F Lin Q Leinonen R Apweiler R Hermjakob H The Protein Identifier Cross-Referencing (PICR) service: reconciling protein identifiers across multiple source databases BMC Bioinformatics 2007 8 401 10.1186/1471-2105-8-401 17945017 PMC2151082

[B17] Pipeline Pilothttp://accelrys.com/products/pipeline-pilot

[B18] PubChem http://pubchem.ncbi.nlm.nih.gov/

[B19] Bolton E Wang Y Thiessen PA Bryant SH PubChem Wheeler RA, Spellmeyer DC Integrated Platform of Small Molecules and Biological Activities Annual Reports in Computational Chemistry 2008 4 Washington, DC: American Chemical Society 12

